# Plexin-B1, glycodelin and MMP7 expression in the human fallopian tube and in the endometrium

**DOI:** 10.1186/1477-7827-7-152

**Published:** 2009-12-29

**Authors:** Michal Amir, Shabtai Romano, Shlomit Goldman, Eliezer Shalev

**Affiliations:** 1 Laboratory for Research in Reproductive Sciences, Department of Obstetrics and Gynecology, Ha'Emek Medical Center, 18101, Afula, Israel; 2 Rappaport Faculty of Medicine, Technion-Israel Institute of Technology, Haifa, Israel

## Abstract

**Background:**

To study the expression of Plexin-B1, Glycodelin, and MMP7 during the menstrual cycle in the endometrium and in the fallopian tube.

**Methods:**

The research included women undergoing hysterectomy, tubal sterilization or salpingo-oophoerectomy. Total RNA from endometrial and fallopian tube tissues was extracted using a total RNA isolation kit. Semi-quantitative RT-PCR was performed to examine mRNA relative expression.

**Results:**

Plexin-B1 expression in the endometrium was significantly higher on days 19 - 23 compared to days 12 - 14 (1.166 +/- 0.42 versus 0.523 +/- 0.299), P < 0.005. In the fallopian tube the level of plexin-B1 did not change significantly throughout the menstrual cycle. Glycodelin expression was significantly higher on days 19 - 23 compared with days 12-14, both in the endometrium (0.819 +/- 0.564 versus 0.072 +/- 0.343, P < 0.05) and the fallopian tube (0.796 +/- 0.196 versus 0.329 +/- 0.398, P < 0.05). Although the level of MMP7 secretion was the highest in the secretory phase the difference from the proliferative phase did not reach statistical significance, neither in the endometrium nor in the fallopian tube. This could result from a lack of power.

**Conclusions:**

In the endometrium, both Glycodelin and Plexin-B1 are exhibiting a cyclic pattern suggesting a possible steroid regulation and a role in endometrial receptivity.

## Background

Implantation in humans involves complex interactions between the embryo and the maternal endometrium. Endometrial receptivity is suggested to be a property of the endometrial epithelial cells. The molecular mechanisms by which the surface of the human endometrium acquires morphological changes, leading to receptive features, are still unclear. Cytokines, growth factors, hormones, extracellular matrix proteins and enzymes, angiogenic factors, cell-cell adhesion molecules and receptors are all involved in this complex process [1-5]. The endometrium is receptive to embryonic implantation during a defined window that is temporally and spatially restricted [1-5]. Coordinated effects of ovarian progesterone and estrogen play critical roles in establishing uterine receptivity for implantation [1-5]

In order to define the proteins involved in the implantation processes, gene expression in the endometrium was investigated during the menstrual cycle [6-8]. The expression of several proteins in the fallopian tube, with comparison to the endometrium, was also described [9-11].

In the present study, we investigated the expression in the endometrium and the fallopian tube of three genes, proposed to be involved in endometrial receptivity and maternal-trophoblastic interactions.

Glycodelin is a member of the lipocalin family of proteins, is synthesized in the endometrium in response to progesterone and relaxin and has immunosuppressive properties and is suggested as a potential receptivity marker [12].

Matrix metalloproteinase 7 (MMP7) is secreted mostly from the endometrial epithelium cells where attachment occurs and is suggested to be important in the implantation process [13].

Plexin B1 is a member of the plexin receptor family for secreted and membrane-bound semaphorines guiding cell migration and exon extension [14]. Plexin B1 is known to be involved in angiogenesis, epithelial morphogenesis and invasive growth in epithelial cells [15]. Recently, Plexin B1 was found to be expressed in the mouse ovary and to have a role in follicular growth and steroidogenesis [16] and in endometrial receptivity of human endometrial cell lines [17].

## Methods

### Patient selection

The research included women undergoing hysterectomy, tubal sterilization or salpingo-oophoerectomy for reasons other than malignant or premalignant disease. All patients undergoing adnexal surgery agreed to endometrial sampling (pipelle) during the procedure. Tubal sterilization was performed by cutting the mid part (ampula) of the tubes. Sixteen women underwent hysterectomy: eight due to myomatous uterus, four due to metrorrhagia, three due to uterine prolapse and one due to chronic pelvic pain. An additional four underwent tubal sterilization and two had a salpingo-oophorectomy due to an ovarian cyst. Patients with endometriosis, inflammatory or infectious disease were excluded. From each participant two samples were taken, one from the endometrium and the second from the fallopian tube.

All included women reported having regular menstrual cycles (28-35 days) and were not pregnant at the time of surgery. All participants were divided according to their phase in the menstrual cycle. Day of menstrual cycle was calculated from the first day of the last menstruation prior to the operation day. Five various phases of the menstrual cycle were determined: early proliferative (day 5-7), mid-proliferative (day 8-11), late proliferative (day 12-14), early secretory (day 15-18) and mid-secretory (day 19-23). Dating of the menstrual cycle phase was confirmed with histology and hormonal profile.

The women's age ranged from 30 to 50 years with mean age of 41.13 ± 5.43 years. Blood samples for hormonal profile, including LH, FSH, Estradiol and Progesterone levels were drawn 2-24 hours before surgery.

In the operation room a small sample of endometrium and from the tubal epithelium was drawn for the research frozen at -80°C until use. The remaining tissue was sent for pathological examination and checked for occult malignancy, signs of inflammation and endometrial dating. If a mismatch was found between menstrual cycle day, histological dating and hormone profile, the participant was withdrawn from the study. A total of 34 women were sampled, but only 22 were included in the study. 12 women were withdrawn from the study as follows: four were withdrawn due to a mismatch in dating, five due to pathological (atrophic or hyperplastic) endometrium and three due to unsatisfactory samples. The 22 patients were divided into 5 groups according to the menstrual phase. There was no significant difference in terms of the patient's age between the five groups (Table 1).

**Table 1:** Characteristics and hormonal profile (Mean ± SD) of the study groups.

Menstrual Phase (days)	Early proliferative (5-7)	Mid proliferative (8-11)	Late proliferative (12-14)	Early Secretory (15-18)	Mid Secretory (19-23)
No. cases	4	4	4	4	6
Age	43.6 ± 0.5	37.5 ± 8.81	41 ± 3.55	41.2 ± 6.8	42.1 ± 4.63
LH (IU/L)	3.66 ± 0.9	6.35 ± 2.15	20.12 ± 21.6	29.25 ± 43.68	2.94 ± 1.92
FSH (IU/L)	5.38 ± 2.33	5.83 ± 3.0	7.1 ± 3.57	6.54 ± 4.62	5.07 ± 7.44
Progesterone (ng/mL)	0.36 ± 0.28	0.42 ± 0.26	0.61 ± 0.55	1.42 ± 0.78	7.57 ± 4.42
E2 (pg/ml)	64.5 ± 25.11	150.5 ± 73.5	179.77 ± 39.1	197.52 ± 156.94	117.67 ± 107.53

The local ethics committee and the National Committee for Human Medical Research granted ethical approval for the study (2830404) and all patients gave informed consent.

### Sample collection

All specimens were collected in aseptic conditions in the operating theatre.

Endometrial samples from the uterus were taken by opening the uterine cavity and peeling the inner layer. Due to their small size, fallopian tube samples were taken complete. Endometrial samples from women undergoing adnexal surgery were taken using a pipelle curette.

Samples were collected at room temperature and transported to the laboratory where they were immediately frozen and kept at -80°C in until used.

### RNA extraction

Total RNA from endometrial and fallopian tube tissues was extracted using a total RNA isolation kit EZ-RNA (Kibbutz Bet HaEmek, Israel) according to manufacturer's instructions.

RNA concentration was determined spectrophotometrically. First strand cDNA synthesis for RT-PCR: To obtain the cDNA, RNA (5 μg) was denatured at 70°C for 10 min and then reverse transcribed in the presence of 25 ng/μl random primer (Promega, Mannheim, Germany), 2.5 mM MgCl2, 0.5 mM deoxy-NTPs, 10 mM dithiothreitol, and 10 U ribonuclease H- reverse transcriptase (SuperScript II RT, Life Technologies, Inc Invitrogen, Dorset, UK.) for 60 min at 42°C, and 5 min at 95°C. Subsequently, 10 μl of the resulting cDNA was used as a template for PCR.

### Reverse transcription-polymerase chain reaction (RT-PCR)

The 1st strand cDNA product, 10 μl, was subsequently amplified in a total volume of 50 μl, containing 1.5 U Taq DNA polymerase (Sigma, St. Louis, MO, USA), 200 μM dNTPs (Promega, Mannheim, Germany), 2 mM MgCl2, 50 pmol of each primer and buffer (10 mM Tris-HCl, 50 mM KCl, 0.1% Triton x-100). Primers for Glycodelin, MMP7, Plexin B1 and GAPDH were used. The PCR amplification for all sets of primers was performed for 30 cycles in an automated thermocycler profile. Before starting, each primer was normalized for sub-saturation conditions. We used a series of dilutions of a bulk RNA preparation to determine sub-saturation conditions for the PCR products using GAPDH. The amplification parameters were denatured at 94°C for 5 minutes, followed by 30 cycles as follows: denaturation at 94°C for 30 seconds, annealing at 58°C for 1 minute and elongation at 72°C for 90 seconds and at the end of program 72°C extension for 10 minutes. RT-PCR products were analyzed by 2.5% agarose ethidium bromide gel electrophoresis. Images were captured with Polaroid film (Hertfordshire, UK) under UV light. Products were quantified using a Densitometer system endowed with TINA software (Raytest, Staubenhardt, Germany). Amplification of the housekeeping gene GAPDH transcripts was performed simultaneously to confirm RNA integrity, efficiency and for quantification of cDNA. Negative control reactions containing samples without cDNA or Taq enzyme were used.

### Primer design

The sequences of appropriate primers were obtained from the gene bank and prepared at IDT Inc., Hy-Labs, Rehovot, Israel.

Reverse transcription-polymerase chain reaction (RT-RCR) for: Glycodelin (gene bank access no. NM_001018049)

FWD 5'-AAGTTGGCAGGGACCTGGCACTC-3'; Glycodelin REV 5'-ACGGCACGGCTCTTCCATCTGTT-3' (420 bp product).

MMP7 (gene bank access no. NM_002423)

FWD 5'-AAACTCCCGCGTCATAGAAAT-3';

MMP7 REV 5'-TCCCTAGACTGCTACCATCCG-3' (394 bp product);

Plexin B1 (gene bank access no. F09621) Plexin B1 FWD 5'-GCAGTGTGTTATCCTTTAATGAAA-3' Plexin B1 REV 5'-CCACTACAAACTGACCCCTC-3'. (150 bp product). For normalization we used the levels of the housekeeping gene GAPDH (gene bank access no. M33197).

GAPDH (FWD 5'-TGATGACATCAAGAAGGTGGTGAAG-3'; REV 5'-TCCTTGGAGGCCATGTGGGCCAT-3' (240 bp product).

### Statistical analysis

Results are expressed as mean ± SEM of 4-6 independent experiments for each group. Statistical analysis was performed using the SPSS statistical software. Due to the small sample size in each group, analysis was done using a nonparametric test: Cruskal-Wallis for each individual parameter. P < 0.05 was considered statistically significant.

## Results

### Glycodelin expression during the menstrual cycle in the human endometrium and fallopian tube

**Figure 1:**
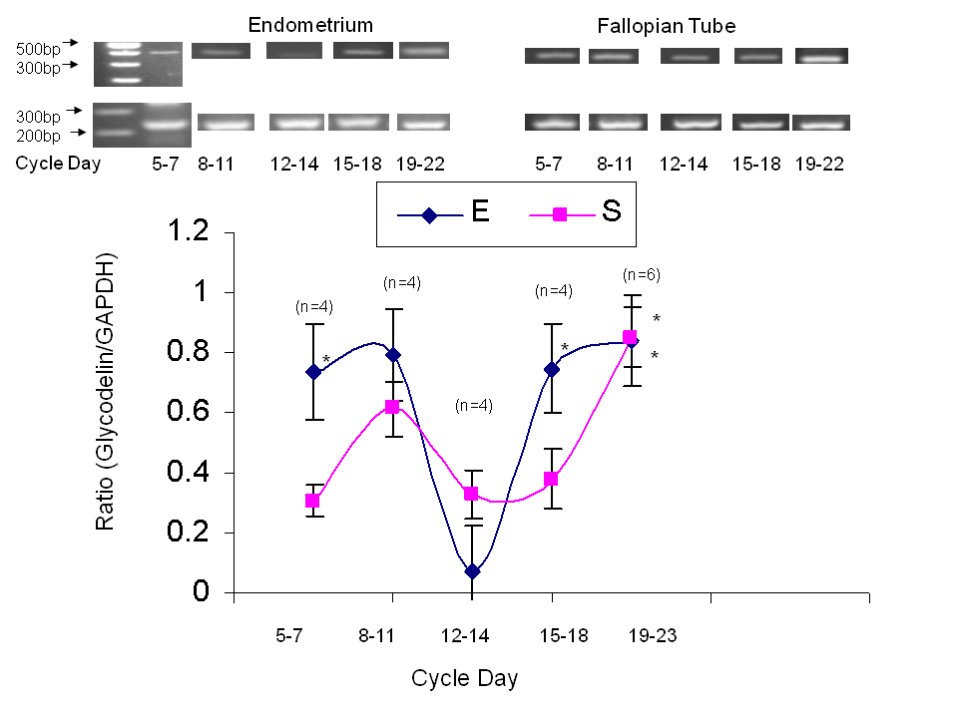
**Glycodelin expression in primary endometrial versus fallopian tube biopsies**. Line-graph representing mean ± SEM of total Glycodelin to GAPDH ratio from 22 endometrial and 22 fallopian tube biopsies, taken from different menstrual cycle days. Blue line-graph represents primary endometrial biopsies, Pink line-graph represents fallopian tube biopsies, * = P < 0.05 in both tissues between days 19-23 compared to days12-14.

A total of 22 endometrial and fallopian tube biopsies were analyzed for Glycodelin expression. Semi-quantitative RT-PCR was performed to examine Glycodelin mRNA relative expression (Table 2, Figure 1). The 420-bp product corresponding to Glycodelin and a 240-bp product for glyceraldehyde-3-phosphate dehydrogenase (GAPDH) as an internal control were detected. To compare Glycodelin mRNA relative expression levels between periods, we analyzed the ratio of each independent experiment between the expression level of both Glycodelin and GAPDH from the same tissue. Figure 1 is a summary of Glycodelin expression through various periods during menstrual cycle. In the endometrium, glycodelin expression declines sharply on days 12-14 as compared with days 5-7 (0.072 ± 0.15 versus 0.734 ± 0.16 Glycodelin/GAPDH ratio, P < 0.05). Glycodelin expression was found to be significantly higher in days 19 - 23 compared to the level in days 12-14 (0.819 ± 0.564 versus 0.072 ± 0.343, P < 0.05). Similar results were observed in the fallopian tube.

**Table 2:** Expression values of PLB1, MMP 7 and Glycodelin (Mean ± SD) in the endometrium (E) and in the fallopian tube epithelium (F).

Menstrual Phase (days)	*Early proliferative (5-7)	*Mid proliferative (8-11)	Late proliferative (12-14)	*Early Secretory (15-18)	*Mid Secretory (19-23)
**PLB1 E**	0.709 ± 0.215 P = 0.406	0.892 ± 0.461 P = 0.227	0.523 ± 0.299	0.881 ± 0.664 P = 0.135	1.166 ± 0.427 P = 0.002
**PLB1 F**	0.446 ± 0.261 P = 0.473	0.822 ± 0.482 P = 0.419	0.580 ± 0.279	0.703 ± 0.241 P = 0.529	0.741 ± 0.127 P = 0.093
**MMP 7 E**	0.285 ± 0.232 P = 0.722	0.629 ± 0.684 P = 0.525	0.346 ± 0.200	0.837 ± 0.627 P = 0.186	0.698 ± 0.402 P = 0.14
**MMP 7 F**	0.302 ± 0.381 P = 0.635	0.338 ± 0.423 P = 0.692	0.207 ± 0.213	0.298 ± 0.286 P = 0.631	0.611 ± 0.458 P = 0.115
**Glycodelin E**	0.742 ± 0.157 P = 0.04	0.791 ± 0.508 P = 0.227	0.072 ± 0.343	0.747 ± 0.502 P = 0.003	0.819 ± 0.564 P = 0.01
**Glycodelin F**	0.235 ± 0.269 0.739	0.612 ± 0.338 P = 0.692	0.329 ± 0.398	0.376 ± 0.492 P = 0.88	0.796 ± 0.196 P = 0.002

* Versus days 12-14

### MMP7 expression during the menstrual cycle in the human endometrium and fallopian tube

**Figure 2:**
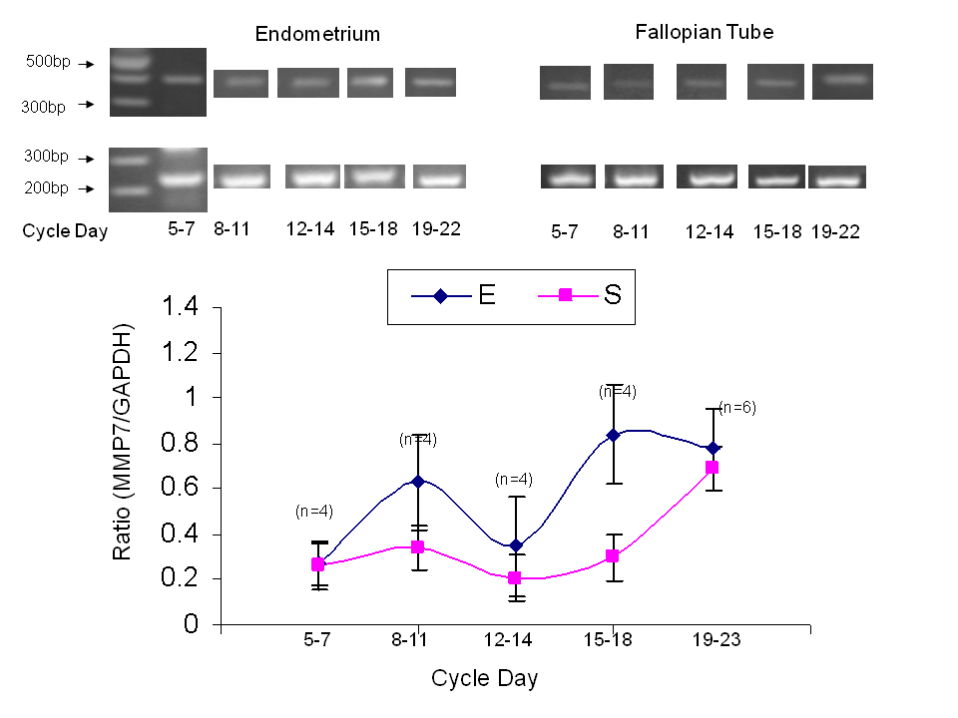
**MMP7 expression in primary endometrial versus fallopian tube biopsies**. Line-graph representing mean ± SEM of total MMP7 to GAPDH ratio from 22 endometrial and 22 fallopian tube biopsies, taken from different menstrual cycle days. Blue line-graph represents primary endometrial biopsies, Pink line-graph represents fallopian tube biopsies.

A total of 22 endometrial and fallopian tube biopsies were analyzed for MMP7 expression (Table 2, Figure 2). Semi-quantitative RT-PCR was performed to examine MMP7 mRNA relative expression. The 394-bp product corresponding to MMP7 and a 240-bp product for glyceraldehyde-3-phosphate dehydrogenase (GAPDH) as an internal control were detected. To compare MMP7 mRNA relative expression levels between periods, we analyzed the ratio of each independent experiment between the expression level of either MMP7 and GAPDH from the same tissue. Figure 2 summarizes MMP7 expression through the various periods during the menstrual cycle. Although the level of MMP7 secretion was the highest in the secretory phase, the difference from the proliferative phase did not reach statistical significance, neither in the endometrium nor in the fallopian tube. This could result from a lack of power.

### Plexin B1 expression during the menstrual cycle, in the human endometrium and in the fallopian tube

**Figure 3:**
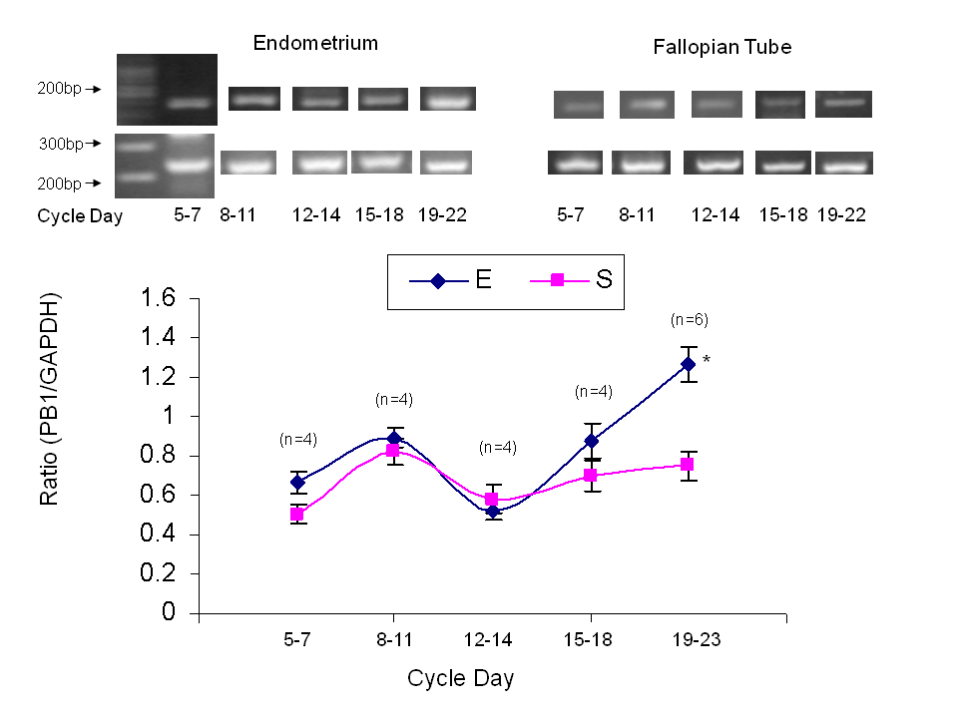
**Plexin B1 expression in primary endometrial versus fallopian tube biopsies**. Line-graph representing mean ± SEM of total Plexin B1 to GAPDH ratio from 22 endometrial and 22 fallopian tube biopsies, taken from different menstrual cycle days. Blue line-graph represents primary endometrial biopsies, Pink line-graph represents fallopian tube biopsies, * = P < 0.05 in the endometrium between days 19-23 compared to days12-14.

A total of 22 endometrial and fallopian tube biopsies were analyzed for plexin B1 expression (Table 2, Figure 3). Semi-quantitative RT-PCR was performed to examine Plexin B1 mRNA relative expression. The 150-bp product corresponding to Plexin B1 and a 240-bp product for glyceraldehyde-3-phosphate dehydrogenase (GAPDH) as an internal control were detected. To compare Plexin B1 mRNA relative expression levels between periods, we analyzed the ratio of each independent experiment between the expression level of either Plexin B1 and GAPDH from the same tissue. Figure 3 summarizes Plexin B1 expression through various periods during the menstrual cycle. Plexin B1 expression in the endometrium during days 19 - 23 was significantly higher compared to days 12 - 14 (1.166 ± 0.42 versus 0.523 ± 0.299, P < 0.005). In the Fallopian tube Plexin B1 level did not change significantly through the menstrual cycle.

## Discussion

The ability of uterine epithelium to allow the trophoblast to adhere depends on the acquisition of a specific functional state referred to as receptivity [1-5]. Epithelial cells have an apical plasma membrane that is normally repellent and does not allow opposing uterine or embryonic cells such as trophoblast to adhere. At the time of implantation, however, uterine epithelial cells are reprogrammed towards adhesiveness for trophoblast [1-5]. It has been proposed that this process may be under the control of master genes, which regulate the expression of the polarized epithelial phenotype and can prepare the apical cell pole of uterine cells contact with the trophoblast [18].

In this study women with an age ranging from 30 to 50 years with regular menstrual cycle were selected. It is well accepted that age is a major fertility rate limiting factor in terms of the ovary and folliculogenesis. However, from the oocyte donation model, it seems that the recipient's age does not play a substantial role in the success of oocyte donation, implying that endometrial receptivity is unaltered by age [19,20].

In agreement with previous studies, both glycodelin [12] and MMP7 [21] genes were expressed in the endometrium and in the fallopian tube. Glycodelin in the fully glycosylated form, a 28-kD glycoprotein, is the predominant protein product of secretory-phase endometrial epithelial cells [12]. Previously it has been referred to as placental protein 14 [12], pregnancy-associated endometrial 2 globulin [12], and progestagen-associated endometrial protein [12]. Although primarily produced in the uterus, this protein has also been detected in follicular and seminal fluids, in cultured hematopoietic precursor cells, and in breast carcinoma. Matrix metalloproteinase-7 (MMP7, matrilysin or uterine metalloproteinase) degrades casein, fibronectin and gelatin types I, III, IV and V [21]. In a previous study, MMP7 has been shown to be expressed during the receptive phase localized to endometrial glandular and luminal epithelium with low transcript levels found in infertile patients [21]. In the present study, the levels of MMP7 during the secretory phase were higher than in the proliferative phase, however this change was not statistically significant and may result from a lack of statistical power.

All three genes were also expressed in the fallopian tube. Given that the fallopian tubes and the uterus are both from the same Müllerian-tract origin, it is not surprising that similar genes are present in both the uterus and the fallopian tubes. Still, the similar pattern of expression should expose the embryo to undesirable ectopic implantation. The fact that this is not usually the case suggests that ectopic implantation is not a passive randomized event but requires certain pathology in the fallopian tube or in the embryo or in both.

In the present study plexin B1 expression was found in human endometrial tissue and its level was found to be significantly higher at the period corresponding with the implantation window. The involvement of the Plexin-Semaphorin system in the reproductive system has been scarcely investigated. Plexin B1 expression was found in the mouse ovary, suggesting a possible role for the receptor in follicular growth and development [16]. Plexin A1, as well as semaphorin 3A, was found in the human placenta and uterus [22]. Plexin B1 was also found in a human endometrial cell line and was suggested to have a role in endometrial receptivity [17]. To the best of our knowledge, this is the first report showing the expression of Plexin B1 in human endometrial tissue. The significantly higher expression level on days 19-23 might suggest that plexin B1 is under hormonal regulation.

## Competing interests

The authors declare that they have no competing interests.

## Authors' contributions

MA participated in the design, carried out the laboratory work and drafted the manuscript. SR coordinated and executed the clinical part of the study. SG participated in the design, coordinated the laboratory work, took part in the analysis and helped in drafting the manuscript. ES conceived and designed the study, analyzed the results and edited the manuscript. All authors read and approved the final manuscript.
